# Racial Variation in Echocardiographic Reference Ranges for Left Chamber Dimensions in Children and Adolescents: A Systematic Review

**DOI:** 10.1007/s00246-018-1873-0

**Published:** 2018-04-04

**Authors:** Edith D. Majonga, Gabrielle Norrish, Andrea M. Rehman, Katharina Kranzer, Hilda A. Mujuru, Kusum Nathoo, Jon O. Odland, Juan P. Kaski, Rashida A. Ferrand

**Affiliations:** 1 London School of Hygiene and Tropical Medicine, London, UK; 2 Biomedical Research and Training Institute, Harare, Zimbabwe; 3 Centre for Inherited Cardiovascular Diseases, Great Ormond Street Hospital, London, UK; 4 Institute of Cardiovascular Science, University College London, London, UK; 5 National Mycobacterial Reference Laboratory, Leibniz Research Centre, Borstel, Germany; 6 Department of Pediatrics, University of Zimbabwe, Harare, Zimbabwe; 7 UiT The Arctic University of Norway, Tromsø, Norway; 8 Department of Public Health, Faculty of Health Sciences, University of Pretoria, Pretoria, South Africa; 9 Clinical Research Department, London School of Hygiene and Tropical Medicine, Keppel Street, London, WC1E 7HT UK

**Keywords:** Echocardiography, Reference ranges, Left ventricle, Left atrium, Children

## Abstract

Echocardiography plays a critical role in the assessment of cardiac disease. Important differences in echocardiographically derived cardiac chamber dimensions have been previously highlighted in different population groups in adult studies, but this has not been systematically studied in children, whose body size changes throughout childhood. The aim of this study was to review the distribution of available reference ranges for the left cardiac chamber dimensions in older children and adolescents. The following electronic data bases were searched: Medline, Embase and Web of Science were searched to identify studies which have established echocardiographic reference ranges of left heart parameters in children and adolescents from 1975 to December 2017. There was no geographical limitation. All results were imported into Endnote. Retrieved articles were screened and data extracted by two independent reviewers. A total of 4398 studies were retrieved, with 36 studies finally included in this review. 29 (81%) references were from North America and European (Caucasians) populations, with only one study each from Africa and South America. Two-dimensional and M-mode techniques were the most commonly used echocardiography techniques. There were methodological variations in techniques and normalisation of references. Comparison of selected cardiac measures showed significant differences for interventricular septal thickness among Black African, Indian, German and US American children. Available echocardiographic references cannot be generalised to all settings and therefore, there is need for locally relevant reference ranges. Africa and South America are particularly under-represented. Future studies should focus on developing comprehensive echocardiographic reference ranges for children from different racial backgrounds and should use standardised techniques.

**Electronic supplementary material:**

The online version of this article (10.1007/s00246-018-1873-0) contains supplementary material, which is available to authorized users.

## Introduction

Echocardiography plays a critical role in the assessment of cardiac structure and function. Due to changes in body size during childhood, the evaluation of cardiac chambers is highly reliant on the availability of reference ranges, the quality of which depends largely on the availability of a representative sample of healthy subjects and the methods employed to collect the data. The definition of what is “normal” varies widely according to age, body surface area (BSA), gender and race [1, 2]. Studies in adults have shown racial differences in echocardiographically derived cardiac chamber dimensions [1]. These differences may be more apparent in children whose body size changes throughout childhood, but this has not been investigated systematically.

The aim of this study was to systematically review racial distribution and methods used in available echocardiographic reference ranges for left ventricular (LV) and atrial (LA) chamber dimensions in children and adolescents. In addition, we compared values of selected chamber dimensions in different racial groups which have utilised the same methods for any differences.

## Materials and Methods

This review was registered with the international prospective register of systematic reviews (PROSPERO; registration number CRD42015026030).

### Type of Studies

All available studies that reported echocardiographic reference ranges for left cardiac chamber dimensions in healthy children and adolescents, regardless of echocardiographic technique, were considered for inclusion in this review.

### Inclusion Criteria and Exclusion Criteria

Studies including at least 50 healthy participants aged 5–21 years that reported echocardiographic measurements at rest were included. We considered studies written in English and published in peer-reviewed journals. Studies that only included neonates and infants, were conducted at high altitude (> 24,000 m above sea level); involved performing cardiac measures during or after exercise; or were based on autopsy specimens were excluded. Systematic reviews and meta-analyses were also excluded.

### Search Strategy

The following electronic databases were searched: Medline, Embase and Web of Science were searched. In addition, reference lists of selected studies and other systematic reviews were manually reviewed to identify other possible studies for inclusion. The search strategy included the following words: “echocardiography” AND “reference values” OR “normative” OR “reference standards” OR “reference intervals” AND “child” OR “children” OR “adolescent” OR “*z* score” (Table 1). Appropriate Boolean operators and truncation were used on synonyms. Both medical subjects and keywords were used. The same search strategy was adapted for all the listed databases. All results were imported into Endnote X7 (Thomas Reuter).

**Table 1:** Search strategy

Concepts	Set	Search terms
Echocardiography	1	echocardiography.mp or exp echocardiography/
References	2	exp reference values/or normative.mp
	3	Reference adj1 values
	4	Reference adj1 standards)
	5	Reference adj1 interval*
	6	Normal values/or reference value/or reference interval*.mp
Children	7	Child.mp or child/
	8	adolescent*.mp or exp adolescent/
	9	pediatric.mp or pediatrics/
	10	Paediatric.mp or paediatrics/
	11	Children*.mp
	12	Infant.mp or exp Infant/
Z-score	13	z-score.mp
	14	2 or 3 or 4 or 5 or 6 or 13
	15	7 or 8 or 9 or 10 or 11 or 12 or 13
	16	1 and 14
	17	15 and 16

Duplicate citations were removed. Titles and abstracts from the search results were screened independently by two reviewers (EDM) and (GN). The full texts of potentially eligible studies were obtained and assessed in duplicate using a standardised checklist. Any disagreements about inclusion of studies were resolved by consensus.

### Data Extraction and Analysis

The following data were extracted using a standard data extraction form: author; population studied; sample size; age range; echocardiography technique; parameters measured, and type of normalisation used.

A two-sample Kolmogorov–Smirnov non-parametric test was used to compare distributions of *z* score = 0 and 2 for selected cardiac measures of studies which used the same technique for performing echocardiography and the same method for calculation of BSA for normalisation. The selected *z* score represents the mean predicted value and the upper cut-off for the normal range for a cardiac measure. The null hypothesis was that the compared groups were sampled from populations with identical distributions. Due to multiple testing, the chance of obtaining a significant *p* value when in fact there is not a true difference between distributions was high. Therefore, we used the Bonferroni adjustment, with a *p* value < 0.005 considered significant.

### Quality of the Studies

We adapted the Newcastle-Ottawa Scale for assessing quality of non-randomised studies to suit cross-sectional studies in the systematic review [3]. In our tool, we assigned scores instead of stars (Table 2). The following criteria were used to determine the quality of the studies: representativeness of the sample; sample size; sample selection; standardisation of image acquisition and statistical methods used.

**Table 2:** Criteria for assessment of quality of studies using the Newcastle-Ottawa Scale adapted for cross-sectional studies

Scale of coding
Representativeness of sample
Truly representative = 3
Somewhat representative = 2
No description of sampling strategy = 1
Sample size
Justified and acceptable = 2
Not justified = 1
Sample selection
Hospital/volunteer = 3
Databases = 2
Not reported = 1
Standardisation of images
American Society for Echocardiography guidelines/other = 2
Not reported = 1
Statistical methods
Rigorous with clear exclusion criteria of abnormal cases = 3
Acceptable = 2
Not appropriate or incompletely described = 1
Total score
11–13 = good quality
8–10 = acceptable
5–7 = poor

## Results

**Fig. 1:**
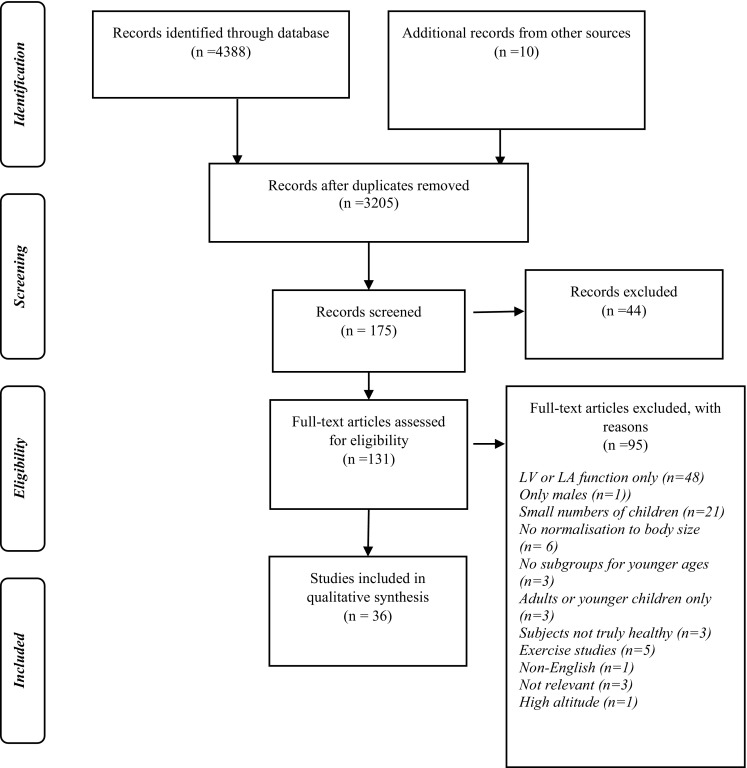
PRISMA flow diagram for process of selecting included studies

A total of 4398 citations were retrieved dating from 1975 to June 2017. Of these, 1193 duplicates were removed, and a further 3075 citations were excluded based on title and abstract (Fig. 1). Full texts of a total of 130 studies were reviewed and 36 studies were included. Characteristics of the included studies are in Table 3.

**Table 3:** Characteristics of studies

Author	Population studied	Sample size	Age range (y)	Technique	Parameters measured	Standardisation of images	Normalisation	Quality of the study
Lopez et al. [4]	US American and Canadian	3215	0–18	2D	LVEDD, IVSd, LVPWd, LV length, LV area, LV volume, LVM	ASE	BSA (Haycock)	Good
Gokhroo et al. [5]	Indian	746	4–15	M mode/2D	LVEDD, LVESD, IVSd, IVSs, LVPWd, LVPWs, LA length	ASE	BSA (Haycock)	Good
Majonga et al. [6]	Zimbabwean (Black)	282	6–16	M mode	LVEDD, LVESD, IVSd, IVSs, LVPWd, LVPWs, LA	ASE	BSA (Dubois)	Good
Foster et al. [7]	US Americans and Canadian	1710	5–18	M mode/2D	LVM	ASE	Lean body mass	Good
Chinali et al. [8]	Italian and German (Caucasians)	400	0–18	M mode/2D	LVM	ASE	Height	Good
Cantinotti et al. [9]	Italian (Caucasians)	1091	0–17	M mode/2D	LVED area, LVES area, LVED length, LVES length, LVEDD, LVESD, LA length, LA area	ASE	BSA (Haycock)	Good
Oran et al. [10]	Turkish	1200	0–17	M mode/2D	LVEDD, LVESD, IVSd, LVPWd, LA	ASE	Weight	Good
Motz et al. [11]	German	9858	0–19	M mode/2D	LVEDD, LVESD, IVSd, LVPWd	ASE	Body length	Acceptable
Bhatla et al. [12]	US American	300	0–18	2D	LA volume	ASE	BSA (Haycock)	Acceptable
Kervancioglu et al. [13]	Turkish	208	0–14	M mode/2D	LVM	ASE	BSA (Dubois)	Acceptable
Taggart et al. [14]	US American	522	0–17	2D	LA volume, LA volume index	ASE	Age	Acceptable
Khoury et al. [15]	US American	2273	0–18	M mode/2D	LVM and LVMI	ASE	Age	Good
Pettersen et al. [16]	US American	782	0–18	2D/M-mode	IVSd, IVSs, LVEDD, LVESD, LVPWd, LVPWs, LA	ASE	BSA (Dubois)	Good
Foster et al. [17]	US American & Canadian	440	0–17	M mode	LVM	ASE	Height	Good
Poutanen et al. [18]	Finnish	169	2–27	M mode/2D/3D	LVM	ASE	BSA (Dubois)	Acceptable
Bonatto et al. [19]	Brazilian (Blacks and whites)	595	0–12	M mode	LVEDD, IVSd, LVPWd, LA, LVM, LVMI	ASE	BSA (Dubois)	Acceptable
Overbeek et al. [20]	Netherlands (Dutch)	587	0–18	M mode	LVEDD, LVESD, IVSd, LVPWd	ASE	Weight	Acceptable
Poutanen et al. [21]	Finnish	169	2–27	3D	LA volumes, LVEDV, LVESV	Other	BSA (Dubois)	Acceptable
Brangenberg et al. [22]	German	150	0–14.5	Acoustic quantification	LV volume and area	ASE	Age/HR	Good
Kampmann et al. [23]	German (Whites)	2036	0–18	M mode	IVSd, IVSs, LVEDD, LVESD, LVPWd, LVPWs, LA	ASE/ESC	BSA (Dubois)	Good
Daubeney et al. [24]	British + Australian	125	0–18	2D	LV inflow, LV area	Other (inner edge to inner edge)	BSA (Boyd)	Good
Daniels et al. [25]	US American (Blacks and Whites)	192	7–17	M mode	LVM	ASE	Height/lean body mass/BSA (NR)	Acceptable
Huwez et al. [26]	British	127	0–19	M mode	IVSd, LVEDD, LVESD, LVPWd, LA, LVM, LV volume	ASE & Penn convention	Age/BSA (Dubois)	Good
Malcolm et al. [27]	US American	904	6–16	M mode	LVM	ASE	Age/height	Good
Nidorf et al. [28]	US American	196	0–18	2D	LV diameter, LV length, LA diameter	Other (inner edge to inner edge)	Height	Acceptable
Vogel et al. [29]	German	95	1–17	2D	LVM, LV volume	ASE	BSA (NR)	Acceptable
Pearlman et al. [30]	US American	268	0–18	2D	LA diameters, LA volume	Other (inner edge to inner edge)	BSA (Dubois)	Acceptable
Pearlman et al. [31]	US American	268	0–18	2D	LV length, LV area	Other (inner edge to inner edge)	BSA (Dubois)	Acceptable
Hanseus et al. [32]	Swedish	120	0–16	2D	LA length, area and width, LV diameter, area and width	Other (trailing edge to leading edge)	BSA (NR)	Acceptable
Daniels at al [33]	US American (Blacks and Whites)	334	6–23	M mode	LVMI	ASE	Height/BSA (Dubois)	Acceptable
Akiba et al. [34]	Japanese	110	0–15	M mode	LVEDD, LVESD, LVPWd, LVPWs, LV volume	Other (inner to inner edge)	BSA (Haycock)	Acceptable
Voogd et al. [35]	Netherlands (Dutch)	432	4–17	M mode	IVSd, IVSs, LVEDD, LVESD, LVPWd, LVPWs, LA	Other (leading edge of echoes)	Weight	Good
Henry et al. [36]	US America	92	0–23	M mode	LVEDD, LVESD, IVSd, IVSs, LVPWd, LVPWs, LA	ASE	Weight/BSA (NR)	Acceptable
Saito et al. [37]	Japanese	301	6–15	M mode	LV muscle volume	Other (Standard & Penn convention)	Age/BSA (West Nomogram)	Acceptable
Henry et al. [38]	US American (Caucasians, Blacks and Oriental)	105	0–23	M mode	IVSd, IVSs, LVEDD, LVESD, LVPWd, LVPWs, LA, LVM	Other	BSA (Dubois)	Acceptable
Epstein et al. [39]	US American (Whites, Blacks, Oriental/East Indians)	205	0–18	M mode	IVSd, IVSs, LVEDD, LVESD, LVPWd, LVPWs, LA	Other	BSA (NR)	Acceptable

*M-mode* motion mode, *2D* two-dimensional, *3D* three-dimensional, *US* United States, *M-mode* motion mode, *BSA* body surface area, *LV* left ventricle, *LVEDD* left ventricular diameter at end-diastole, *LVESD* left ventricular diameter at end-systole, *LVEDV* left ventricular volume at end-diastole, *LVESV* left ventricular volume at end-systole, *IVSd* interventricular septum at end-diastole, *IVSs* interventricular septum at end-systole, *LVPWd* left ventricular posterior wall at end-diastole, *LVPWs* left ventricular posterior wall at end-systole, *LVM* left ventricular mass, *LVMI* left ventricular mass index, *LA* left atrium, *HR* heart rate, *NR* not reported

Sixteen (44%) studies were conducted in North America, followed by Europe (*n* = 13, 36%) and Asia (*n* = 5, 14%). Only one study each was conducted in South America (Brazil) and Africa (Zimbabwe). Nine studies reported the race of children studied [6, 8, 9, 19, 23, 25, 33, 38, 39]. Sample sizes of the studies ranged from 95 to 9858. The quality of the studies was good (*n* = 16) or acceptable (*n* = 20) in all cases.

M-mode and/or two-dimensional (2D) echocardiographic techniques were used in 33 (92%) studies. One study used three-dimensional (3D) echocardiography in addition to M-mode and 2D, another utilised 3D only and a third study used a rarely practiced echocardiographic technique called acoustic quantification [18, 21, 22]. Anthropometric and non-anthropometric measures were used for normalising the results: body surface area (BSA) in 18 studies, height (*n* = 4) [8, 11, 17, 28], weight (*n* = 3) [10, 20, 35], age (*n* = 2) [14, 15], and lean body mass (*n* = 1) [7]. The remaining studies used age and heart rate, (*n* = 1); [22] age and BSA, (*n* = 2); [26, 37] age and height, (*n* = 1); [27] height and BSA, (*n* = 1); [33] weight and BSA, (*n* = 1) [36] and one study used height, BSA and lean body mass [25]. Varying methods to calculate the BSA were used: 12 (33%) studies used the Dubois and Dubois method, and 4 (14%) studies used the Haycock. Daubeney et al. used the Boyd method; [24] Saito et al. calculated BSA using the West Nomogram [37] and five studies did not report the method used for calculating BSA [25, 29, 32, 39]. 23 (64%) studies standardised images and performed measurements according to recommendations by the American Society of Echocardiography (ASE); one study used ASE and European Society of Cardiology recommendations and another study used ASE and Penn convention [23, 26]. The remaining studies used other methods of performing measurements, including inner edge to inner edge method in five studies [24, 28, 30, 31, 34]; trailing edge to leading edge in one study [32]; leading edge to leading edge in one study [35]; standard and Penn convention in one study [37]. Three studies described how they measured parameters without stating a specific convention [21, 38, 39].

### Left Ventricular Dimensions

The following LV dimensions were reported: diameter at end-diastole (LVEDD) and/or end-systole (LVESD), posterior wall and interventricular septal, area, length, volume, LV mass and index. Saito et al. developed references for LV muscle volume, which is a rarely used measure in clinical practice [37]. 13 studies were from North American children; 12 studies among European children; five studies from Asia and one study each from South American and African children (Table 3).

### Left Atrium Dimensions

LA diameter references were established in twelve studies, six of which were conducted in US American children [16, 28, 30, 38, 39]. LA length references were from Indian, Italian and Swedish children and of these, two studies also reported data on LA area [5, 9, 32]. LA volume was derived in four studies and three of these were in US American children (Table 3) [12, 14, 21, 30].

### Comparison of LV and LA Dimension Between Studies

Reference values of selected cardiac measures from different racial groups which used M-mode technique and normalised results with BSA were identified and compared (Tables 4, 5). The *p* values for the compared references are shown in Supplementary Tables 1 and 2 and these are for *z* score = 0 and + 2 distributions. The graphical representations of the distributions for the selected cardiac measures are shown in Supplementary Figs. 1 and 2. Mean left ventricular diameter at end-diastole (LVEDD) among US American children and German children was similar (*p* = 0.906). On the other hand, Zimbabwean children had thicker mean interventricular septum at end-diastole (IVSd) than German children had (*p* < 0.001) while US American children and German children were similar (*p* = 0.281). Mean LA diameter was also similar between British and German children (*p* = 0.699).

**Table 4:** A comparison of published echocardiographic normal references for LV dimensions in studies using M-mode and normalised to BSA (for a child with 1 m^2^ BSA)

Author	Population studied	Method for BSA	LVEDD(mm)	LVESD (mm)	IVSd (mm)	IVSs (mm)	LVPWd (mm)	LVPWs (mm)
Gokhroo et al. [5]	Indian	Haycock	35.02 (27.02–42.04)	21.32 (13.81–28.84)	7.4 (5.5–9.3)	11.0 (8.11–13.9)	7.2 (5.4–9.1)	10.8 (10.1–11.5)
Cantinotti et al. [9]	Italian	Haycock	37.86 (31.56–45.42)	22.97 (17.46–30.20)				
Majonga et al. [6]	Zimbabwean	Dubois	37.10 (32.43–41.76)	25.29 (20.84–29.74)	7.0 (5.0–9.1)	9.2 (6.7–11.6)	6.8 (5.2–8.5)	9.0 (6.5–11.4)
Pettersen et al. [16]	US American	Dubois	39.09 (32.06–48.79)	25.1 (19.6–32.1)	5.9 (3.9–9.0)	8.6 (6.0–12.3)	5.4 (3.7–7.9)	10.3 (7.7–13.9)
Kampmann et al. [23]	German	Dubois	38.50 (31.70–45.30)	24.4 (18.6–30.2)	5.8 (4.0–7.6)	8.4 (5.1–11.7)	5.9 (3.7–8.1)	9.5 (6.8–12.2)
Huwez et al. [26]	British	Dubois	38.27 (33.05–43.49)	24.28 (19.79–28.77)	7.1 (5.2–7.1)		6.4 (4.6–8.3)	

Dimensions are mean (± 2SD)

*US* United States, *M-mode* motion mode, *BSA* body surface area, *LV* left ventricle, *LVEDD* left ventricular diameter at end-diastole, *LVESD* left ventricular diameter at end-systole, *IVSd* interventricular septum at end-diastole, *IVSs* interventricular septum at end-systole, *LVPWd* left ventricular posterior wall at end-diastole, *LVPWs* left ventricular posterior wall at end-systole, *LVM* left ventricular mass

**Table 5:** A comparison of published echocardiographic normal references for LA dimensions in studies using M-mode and normalised to BSA (for a child with 1 m^2^ BSA)

Author	Population studied	Technique	Method for BSA	LA diameter (mm)
Majonga et al. [6]	Zimbabwean (Blacks)	M-mode	Dubois	24.05 (19.19–28.91)
Huwez et al. [26]	British	M-mode	Dubois	25.9 (20.3–31.6)
Kampmann et al. [23]	German (Caucasian)	M-mode	Dubois	25 (19.2–30.8)

Dimensions are mean (± 2SD)

*US* United States, *M-mode* motion mode, *BSA* body surface area, *2D* two-dimensional, *LA* left atrium, *A4C* apical four chamber view

Comparison using predicted values of *z* score = +2 (cut-off for upper limit of normal), significant differences were noted in the IVSd measures between Zimbabwean and German children (*p* = 0.001). IVSd measures between German and US American children were significantly different (*p* < 0.001). No significant differences were noted on the distributions of LA measures between the compared studies. The predicted values of *z* score = +2 by Kampmann et al. progressed in a step-wise fashion so for example, a value of IVSd > 10.4 mm had a *z* score = +2 for BSA between 1.7 and 1.9 m^2^. The shape of Kampmann’s distribution for *z* score = +2 was therefore strikingly different from the other references [23].

## Discussion

Accurate assessment of left cardiac chamber size in children relies on the availability of representative reference ranges. In this study, we have systematically reviewed differences between reference ranges for LV and LA chamber dimensions in children and adolescents. We found many studies which have established reference ranges for LV and LA chamber dimensions, reflecting the significant interest in, and importance of, establishing reference ranges for chamber dimensions in children. Most of the reference ranges in children were, however, developed in European and North American (US populations) and mainly in Caucasians. Notably, there was only one study each from South America (Brazil) and Africa (Zimbabwe) and the latter was published very recently, implying that most African countries rely on Western references in clinical practice which may not accurately represent black African children [6, 19].

Comparability of the available reference ranges was limited due to substantial methodological variations, including parameters of normalisation; technique of image acquisition (e.g. 2D or M-mode); measured chamber dimension and/or method used for performing the measurements (e.g. ASE guidelines or other). Cantinotti et al. highlighted that it is imperative to standardise methods of image acquisition and consistency in normalisation of the references [40].

However, we were able to compare a few studies which used similar methods from different races for selected cardiac measures. There were notable differences in some of the measures, particularly interventricular septal thickness among Zimbabwean, German and US American children. Differences between Zimbabwean and German children were consistently demonstrated at both mean-level and upper cut-off for normal distributions. Although no interventricular septal thickness difference was found in the mean distribution of German and US American children, it was evident in the predicted values *z* score = +2. In practice, it is the upper cut-off for normal which is used to define abnormality rather than the mean. Our findings suggest that differences in reference ranges between different racial groups do exist but may be overlooked because of the scarcity of data e.g. African children. The use of inappropriate reference ranges may result in either under- or over-diagnosis of cardiac abnormalities or missing of early cardiac chamber remodelling due to cardiac disease [41]. This highlights the importance of using racial-specific reference ranges in clinical practice.

In a recently published study on effect of age, sex, race and ethnicity in echocardiographic *z* scores of children, significant effects by all the four parameters were observed on *z* scores. However, the authors concluded that these were not of clinical significance [4]. Given that this study was conducted mainly in US American and Canadian children, findings cannot be generalisable to the rest of the world due to non-standardisation of methods in the available references. There are also other geographical confounders such as nutrition and altitude which may affect cardiovascular development [4].

Most studies used the M-mode or 2D echocardiography techniques. Advanced techniques such as 3D echocardiography, which may overcome some of the technical challenges of angle dependence and other geometric assumptions associated with conventional techniques, should be used to more accurately quantify chamber sizes and development of reference ranges [42].

This study is limited by the fact that many of the studies did not report the actual race of the children, and we therefore made assumptions based on the country the study was conducted in. We compared very broad racial groups due to scarcity of data. In addition, we were unable to compare references for other cardiac measures due to varying methods used in the studies. However, in the few selected references and cardiac measures where this was possible, we were able to demonstrate significant differences in different races. We also showed similarities in same racial groups. It is also highly likely that in addition to the varying methods used in other studies, racial differences are also present.

## Conclusion

This review underlines the importance of using race-specific reference ranges for children, as well as the need for standardising echocardiographic methods in deriving those reference ranges. Furthermore, these reference ranges need to be comprehensive, including a wide range of cardiac measures, as some studies only reported normal values for a single cardiac measure. Future studies should focus on including 3D parameters in addition to 2D and M-mode.

## Electronic supplementary material

Below is the link to the electronic supplementary material.

Supplementary material 1 (DOCX 139 KB)
