# Supplementary material for: Racial Variation in Echocardiographic Reference Ranges for Left Chamber Dimensions in Children and Adolescents: A Systematic Review
Source: Pediatr Cardiol. 2018 Apr 4;39(5):859–68. doi: 10.1007/s00246-018-1873-0 (PMC5958170; doi:10.1007/s00246-018-1873-0)
Supplement: Supplementary file 1 — Supplementary material 1 (DOCX 139 KB) [file 246_2018_1873_MOESM1_ESM.docx]

**Supplementary Figure 1:** **A comparison of distributions (Z-score=0) of echocardiographic references for LV dimensions in studies using M-mode technique and BSA normalisation**

Z-score=0 is representing the predicted mean value

*Abbreviations: LVEDD, left ventricular diameter at end diastole; LVESD, left ventricular diameter at end systole; IVSd, interventricular septum at end diastole; IVSs, interventricular septum at end systole; LVPWd, left ventricular posterior wall at end diastole; LVPWs, left ventricular posterior wall at end systole*

**Supplementary Figure 2:** **A comparison of distributions (Z-score=2) of echocardiographic references for LV dimensions in studies using M-mode technique and BSA normalisation**

Z-score=0 is representing the predicted mean value

*Abbreviations: LVEDD, left ventricular diameter at end diastole; LVESD, left ventricular diameter at end systole; IVSd, interventricular septum at end diastole; IVSs, interventricular septum at end systole; LVPWd, left ventricular posterior wall at end diastole; LVPWs, left ventricular posterior wall at end systole; LA, left atrium*

**Supplementary Table 1: Kolmogorov Smirnov test of equality of distribution, p-values comparing references for each of the following variables for zscore=0**

| Reference | Reference | LVEDD  (p-value) | LVEDS  (p-value) | IVSD  (p-value) | IVSS  (p-value) | LVPWD  (p-value) | LVPWs  (p-value) | LA diameter  (p-value) |
| --- | --- | --- | --- | --- | --- | --- | --- | --- |
| Majonga [4] | Kampmann **[8**] | 0.468 | 0.699 | **0.000*** | **0.001*** | 0.016 | 0.699 | 0.994 |
| Majonga [4] | Huwez **[25]** | 0.468 | 0.906 | 1.000 |  | 0.994 |  | 0.699 |
| Majonga [4] | Petterson **[35]** | 0.468 | 0.699 | 0.037 | 0.468 | **0.002*** | 0.468 |  |
| Kampmann [8] | Huwez **[25]** | 1.000 | 0.994 | **0.000*** |  | 0.078 |  | 0.699 |
| Kampmann [8] | Petterson **[35]** | 0.906 | 0.906 | 0.281 | 0.078 | 0.281 | 0.468 |  |
| Huwez [25] | Petterson **[35**] | 0.906 | 0.699 | 0.016 |  | 0.037 |  |  |
| Gokhroo [36] | Cantinotti **[6]** | 0.281 | 0.037 |  |  |  |  |  |

*****, significance at p <0.005

Compared references used the same M-mode technique and BSA normalisation (from Table 4 and 5).

Z-score=0 is representing the predicted mean value

*Abbreviations: LVEDD, left ventricular diameter at end diastole; LVESD, left ventricular diameter at end systole; IVSd, interventricular septum at end diastole; IVSs, interventricular septum at end systole; LVPWd, left ventricular posterior wall at end diastole; LVPWs, left ventricular posterior wall at end systole*

**Supplementary Table 2: Kolmogorov Smirnov test of equality of distribution, p-values comparing references for each of the following variables for Z-score= 2**

| Reference 1 | Reference 2 | LVEDD  (p-value) | LVEDS  (p-value) | IVSD  (p-value) | IVSS  (p-value) | LVPWD  (p-value) | LVPWs  (p-value) | LA diameter  (p-value) |
| --- | --- | --- | --- | --- | --- | --- | --- | --- |
| Majonga [4] | Kampmann **[8]** | 0.281 | 0.906 | **0.001*** | 0.155 | 0.468 | 0.155 | 0.699 |
| Majonga [4] | Huwez [**25]** | 0.281 | 0.906 | 0.994 |  | 1 |  | 0.468 |
| Majonga [4] | Petterson **[35]** | 0.281 | 0.155 | 0.281 | 0.281 | 0.016 | 0.155 |  |
| Kampmann [8] | Huwez [**25]** | 0.906 | 0.906 | **0.002*** |  | 0.699 |  | 0.281 |
| Kampmann [8] | Petterson [**35]** | 0.699 | 0.078 | **0.000*** | **0.002*** | 0.078 | 0.281 |  |
| Huwez [25] | Petterson **[35]** | 0.994 | 0.155 | 0.078 |  | 0.078 |  |  |
| Gokhroo [36] | Cantinotti **[6]** | 0.078 | **0.016**** |  |  |  |  |  |

*****, significance at p <0.005

**, significance at p <0.05

Compared references used the same M-mode technique and BSA normalisation (from Table 4 and 5)

Z-score=2 is representing the upper cut off for normal

*Abbreviations: LVEDD, left ventricular diameter at end diastole; LVESD, left ventricular diameter at end systole; IVSd, interventricular septum at end diastole; IVSs, interventricular septum at end systole; LVPWd, left ventricular posterior wall at end diastole; LVPWs, left ventricular posterior wall at end systole*
